# Glycyrrhetinic Acid Protects Renal Tubular Cells against Oxidative Injury via Reciprocal Regulation of JNK-Connexin 43-Thioredoxin 1 Signaling

**DOI:** 10.3389/fphar.2021.619567

**Published:** 2021-02-02

**Authors:** Yao Zhou, Leiping Gao, Ping Xia, Jing Zhao, Wei Li, Yufeng Zhou, Qingxue Wei, Qijing Wu, Qi Wu, Dongdong Sun, Kun Gao

**Affiliations:** ^1^ Department of Pathophysiology, Xuzhou Medical University, Xuzhou, China; ^2^ Division of Nephrology, Affiliated Hospital of Nanjing University of Chinese Medicine, Nanjing, China; ^3^ Changshu Hospital Affiliated to Nanjing University of Chinese Medicine, Changshu, China; ^4^ School of Integrated Chinese and Western Medicine, Nanjing University of Chinese Medicine, Nanjing, China

**Keywords:** connexin 43, thioredoxin 1, oxidative stress, renal tubular cell, glycyrrhetinic acid

## Abstract

**Background and Objective:** The incidence of chronic kidney disease (CKD) is steadily increasing. Although renal tubular epithelium injury is closely correlated with the prognosis of CKD, the underlying mechanism is not fully understood and therapeutic strategies are limited. The main bioactive component of the Chinese medicine herb, glycyrrhiza, is 18α-glycyrrhetinic acid (Ga), which is also a pharmacological inhibitor of gap junctions. Our previous studies indicated that Ga is able to ameliorate renal cell injury. The present study explored the regulatory role of Ga in redox signaling in renal tubular epithelial cells with oxidative injury.

**Methods:** Rat renal tubular epithelial cells, NRK-52E, were incubated with Px-12, a thioredoxin inhibitor, to mimic thioredoxin deficiency and induce oxidative injury *in vitro*. A Cell Counting Kit-8 was used to analyze cell viability while a reactive oxygen species (ROS)/superoxide (O_2_^−^) fluorescence probe was employed to determine oxidative stress. Apoptosis was evaluated using DT-mediated dUTP nick end labeling/4,6-diamidino-2-phenylindole staining and cleaved caspase 3 protein analysis. Western blot analysis was used to analyze the expression of specific proteins while siRNA transfection was performed to downregulate targeted proteins.

**Results:** Inhibition of thioredoxin 1 by Px-12 triggered renal tubular cell oxidative injury as evidenced by morphological change, loss of cellular viability, over production of ROS and O_2_^−^, and appearance of cleaved caspase-3. Ga significantly attenuated cell oxidative injury, as indicated by the parameters mentioned above. Px-12 induced phosphorylation of c-Jun N-terminal kinase (JNK) and subsequently the expression of connexin 43 (Cx43) in NRK-52E cells. Ga and the JNK inhibitor, sp600125, markedly suppressed Px-12-induced generation of intracellular ROS and O_2_^−^. Inhibition of JNK improved Px-12-elicited NRK-52E cell injury. Moreover, sp600125 inhibited Cx43 expression. After downregulation of Cx43 via Cx43 siRNA transfection, the phosphorylation of JNK was markedly reduced. Furthermore, Ga restored the expression of thioredoxin 1 inhibited by Px-12.

**Conclusion:** ROS-JNK-Cx43-thioredoxin 1 signaling plays a crucial role in renal tubular cell injury. JNK is involved in the regulation of thioredoxin 1 and Cx43, and Cx43 reciprocally regulates thioredoxin 1. Inhibition of gap junctions by Ga alleviated renal tubular oxidative injury via improvement of thioredoxin 1-mediated redox signaling.

## Introduction

The prevalence, health, and financial burden of chronic kidney disease (CKD) has been increasing for decades (Chen et al., 2019a), and in China, the incidence of is 10.8% (Zhang et al., 2012). Advanced CKD progresses into end stage renal disease (ESRD) (Zha and Qian, 2017). Tubular cell injury, such as proteinuria, hypertension, diabetes mellitus, oxidative stress, and inflammation accelerates the progression of CKD to ESRD (Nakagawa et al., 2015; Fujigaki et al., 2017; Valiño-Rivas et al., 2019) and is closely correlated with the prognosis of CKD (Said et al., 2015). Methods to protect renal tubular cells against multiple insults have attracted much attention. Oxidative stress is one of the common pathways of various factors involved in tubular cell damage (Honda et al., 2019) and is thought to be the most important mechanism underlying tubular cell damage (Ogura et al., 2020). However, to date, the mechanism that regulates the process of tubular injury is not fully understood, especially in redox signaling.

Thioredoxin is a small molecular weight protein widely expressed in organisms that not only regulates the redox state of cells, but also functions as a regulator of cytokine activity, protein-binding activity, and apoptosis (Park et al., 2020). The cysteines in the thioredoxin active center can be both in the oxidative form (-S-S-) and reduced form (-SH, -SH), demonstrating their reversible oxidation-reduction characteristics (Namba et al., 2016). Thioredoxin 1 is mainly found in the cytoplasm and thioredoxin 2 is located in mitochondria (Léveillard and Aït-Ali, 2017). Thioredoxin interacting protein (Txnip) binds to thioredoxin 1 to inhibit its reductive activity (Sullivan et al., 2018). Txnip and thioredoxin 1 bind to each other to form the redoxisome and play a vital role in multiple biological processes, especially in redox signal transduction (Nasoohi et al., 2018).

Our previous study showed that the gap junction is involved in anti-oxidative stress and regulation of Txnip in CKD (Gao et al., 2017); whether the gap junction regulates thioredoxin in kidney is unknown. The gap junction is a channel-like structure at the membrane surface of two neighboring cells formed by two docking connexions and plays a vital role in intercellular communication and small molecule interchange (Beyer and Berthoud, 2018). Each connexon consists of six homogenous or heterogeneous units of the gap junction protein, connexin (Stylianaki et al., 2019). Currently, more than 20 kinds of gap junction proteins have been identified (Wang et al., 2018) and are widely present in human cells with channel-dependent and -independent functions (Chen et al., 2019b). Connexin 43 (Cx43) is abundantly expressed in kidney, and functions importantly in CKD via regulating a variety of processes (Price et al., 2020). There is high Cx43 expression in animal kidney tissues subjected to acute kidney injury, and the mechanism may be related to oxidative stress-induced kidney injury (Wang and Tong, 2015). When Cx43 expression is inhibited, the level of oxidative stress in kidney tissue is significantly reduced, and the degree of kidney injury is also markedly alleviated (Prakoura et al., 2018). In gap junction research, 18α-glycyrrhetinic acid (Ga) is a classic pharmacological inhibitor of the gap junction, and has wide pharmacological effects (Rahman et al., 2016). It is also the main bioactive component of glycyrrhiza, one of the frequently used Chinese medicine herbs (Pastorino et al., 2018). Our previous studies have reported that Ga alleviates kidney injury (Yan et al., 2012; Gao et al., 2015). However, the role and mechanism of Ga in tubular cell injury and redox signaling is not fully understood.

We have reported that c-Jun N-terminal kinase (JNK) mediates the induction of Px-12 and Cx43 in tumor cells (Li et al., 2016). Px-12 is a thioredoxin inhibitor. JNK is one of the classical signal transduction pathways belonging to the mitogen activated protein kinase (MAPK) families and is mainly activated by oxidative stress (Dai et al., 2018). The activated JNK signal then promotes cell apoptosis (Guo et al., 2018). It is possible that JNK is involved in the regulation of Cx43 in thioredoxin 1 in kidney.

Since inhibition of Cx43 aggravates renal injury by regulating oxidative stress, and thioredoxin 1 is one of the important regulators of oxidative stress (Gao et al., 2015b), whether and how Cx43 regulates thioredoxin 1 remains unclear. In the present study, Px-12 was used to construct a cell oxidative injury model to investigate the function and mechanism of Cx43 in renal tubular cell oxidative injury.

## Materials and Methods

### Reagents and Antibodies

The Cell Counting Kit-8 (CCK-8, FH783) was obtained from Japan Dojindo Company. The Cellular ROS/Superoxide detection assay kit was obtained from Abcam Reagent Company Limited (Shanghai, China). Phospho-stress-activated protein kinase (SAPK)/JNK (Thr183/Tyr185) (81E11) rabbit mAb (*p*-JNK, 4668), SAPK/JNK antibody (JNK, 9252), phospho-p38 MAPK (Thr180/Tyr182, 9212) antibody, phosphorylated -extracellular signal-regulated kinase (ERK1/2, phosphor-p44/42 MAPK, Thr202/Tyr204, 4376) antibody, caspase-3 antibody (caspase-3, 9662S), *β*-tubulin (9F3) rabbit antibody (β-tubulin, 2128), poly (ADP-ribose) polymerase (PARP) (46D11) rabbit antibody (PARP, 9532S), and thioredoxin 1 (C63C6) rabbit antibody (thioredoxin 1, 2429S) were purchased from Cell Signaling Technology Company Limited (Shanghai, China). Anti-connexin 43 antibody produced in rabbit (Cx43, SAB4501175) and 18α-glycyrrhetinic acid (18α-Ga, G8503) were purchased from Sigma Aldrich China Company. 4,6-diamidino-2-phenylindole (DAPI, KGA215-50) was obtained from Jiangsu KeyGEN BioTECH Corp., Ltd. DT-mediated dUTP nick end labeling (TUNEL, 11684817910) was purchased from Roche Life Science Company. Px-12 (sc-358518) was purchased from Santa Cruz Biotechnology Company. The JNK-mitogen-activated protein kinase (MAPK) signaling pathway inhibitor (sp600125, A4604) was obtained from ApexBio Technology China. The Hiperfect transfection reagent, Cx43 siRNA, and control siRNA were purchased from Qiagen (Qiagen, Hilden, Germany).

### Evaluation of Cell Viability Using the CCK-8 Kit

Cell viability was tested using the CCK-8 reagent kit. In brief, NRK-52E cells (1×10^5^ cells/well) were seeded into a 96-well microplate, then grown in complete Dulbecco’s modified Eagle medium/F12 culture media containing 5% fetal bovine serum for 24 h. The cultivated cells were assigned to different groups (control group, Px-12 treatment group) and incubated with different drugs (18α-Ga, inhibitor) for 24 h. Following addition of CCK-8 working solution and incubation for 1.5 h, the optical density (OD) was measured at 450 nm. Cell viability was represented as a percentage of the control.

### Determination of ROS/O_2_^−^ Production

Intracellular ROS and O_2_^−^ were determined using the ROS/O_2_^−^ reagent kit (Enzo Life Sciences, New York, United States). Cells were evenly transferred to six-well microplates at a density of 1 × 10^6^ cells/ml and cultivated with complete Dulbecco’s modified Eagle medium/F12 culture media containing 5% fetal bovine serum for 24 h. The cells were then assigned to control group, Px-12 treatment group, 18α-Ga treatment group, 18α-Ga and Px-12 co treatment group, inhibitor treatment group, inhibitor and Px-12 co treatment group. 18α-Ga and inhibitor are added 1 h advance. Following another 1 h of incubation, we wash cells twice with PBS. Then 500 µl/well working solution was added to the microplate for the determination of ROS (green) and O_2_^−^ (orange). The mixture was incubated in the dark for 30 min, washed with PBS twice and then photographed with an inverted fluorescence microscope. The fluorescence intensity was measured at 480 nm excitation for green and 550 nm for orange.

### Western Blotting

Cells were grouped, treated with different drugs for a specific time, and then total protein was extracted by SDS lysis buffer together with freshly added proteinase inhibitor cocktail. Lysates were incubated in ice for 15 min with intermittent mixing and then centrifuged at 12,000 rpm for 15 min at 4°C. Total cellular protein concentration was measured using the bicinchoninic acid protein assay kit and separated by 7.5, 10, or 12% SDS-polyacrylamide gels.20 µg or 60 µg total protein was loaded into each well for electrophoresis at 80 V. When the protein moved to the junction of separation gel and concentrated gel, the electrophoresis was changed to 120 V. A 0.22 µm polyvinylidene difluoride membrane was used for electric transfer (250 mA) of proteins to the membrane for 1.5 h, blocked with 5% nonfat milk for 45 min, and then incubated with the primary antibody at 4°C overnight. The membrane was washed three times with PBST and incubated for 1 h with the secondary antibody at room temperature. The membrane was washed again, and images were captured using a chemical imaging system (Shanghai Tanon Technology Co., Ltd., Tanon 4600SF).

### TUNEL/DAPI Staining

Cell-attached slides in the six-well microplate were treated using the same method for the CCK-8 cell viability test. The slides were washed thrice with phosphate-buffered saline (PBS), fixed in 4% polyformaldehyde for 15 min, and then washed thrice with PBS again. The membrane was treated with Triton-X for 2 min, washed thrice with PBS, and then the cell-attached slides were removed. Moisture on the slides was removed, and then dUTP-TdT (9:1) was added to cover the cells. The cell-attached slides were placed back into the six-well microplate, incubated at 37°C in a water bath chamber for 1 h, and then washed thrice with PBS. The slides were incubated in the dark with instant DAPI solution for 5 min, washed thrice with PBS, and dried via centrifugation. After that, anti-fade mounting medium was added, and the cell-attached slides were covered with glass coverslips. The slides were air dried and photographed using a fluorescence microscope.

### Cx43 siRNA Transfection

Cell counts were measured after subculture, the cell density was adjusted to 1 × 10^6^ cells/ml in a six-well microplate, and each well was filled with 2 ml of medium. Then, 24 h after cell adherence, cells were transiently transfected with siRNA specifically targeting Cx43, or a negative control siRNA at a final concentration of 100 nM using HiPerFect transfection reagent. Transfected cells were then either left untreated or exposed to various stimuli. Cells were harvested 24 h after transfection and re-inoculated into a six-well plate for further studies.

### Statistical Analysis

All metric data are expressed as the mean ± standard deviation (SD). Between-group differences were analyzed using a *t*-test. Multiple-group differences were analyzed using analysis of variance (ANOVA), followed by Dunnett’s test. All data were analyzed using IBM SPSS Statistics 23.0 software (IBM SPSS, United States). *p <* 0.05 represents a significant difference. Image analysis was performed using ImageJ v1.46 developed by National Institutes of Health (NIH).

## Results

### Inhibition of Thioredoxin 1 Initiated Renal Tubular Cell Oxidative Injury

**FIGURE 1:**
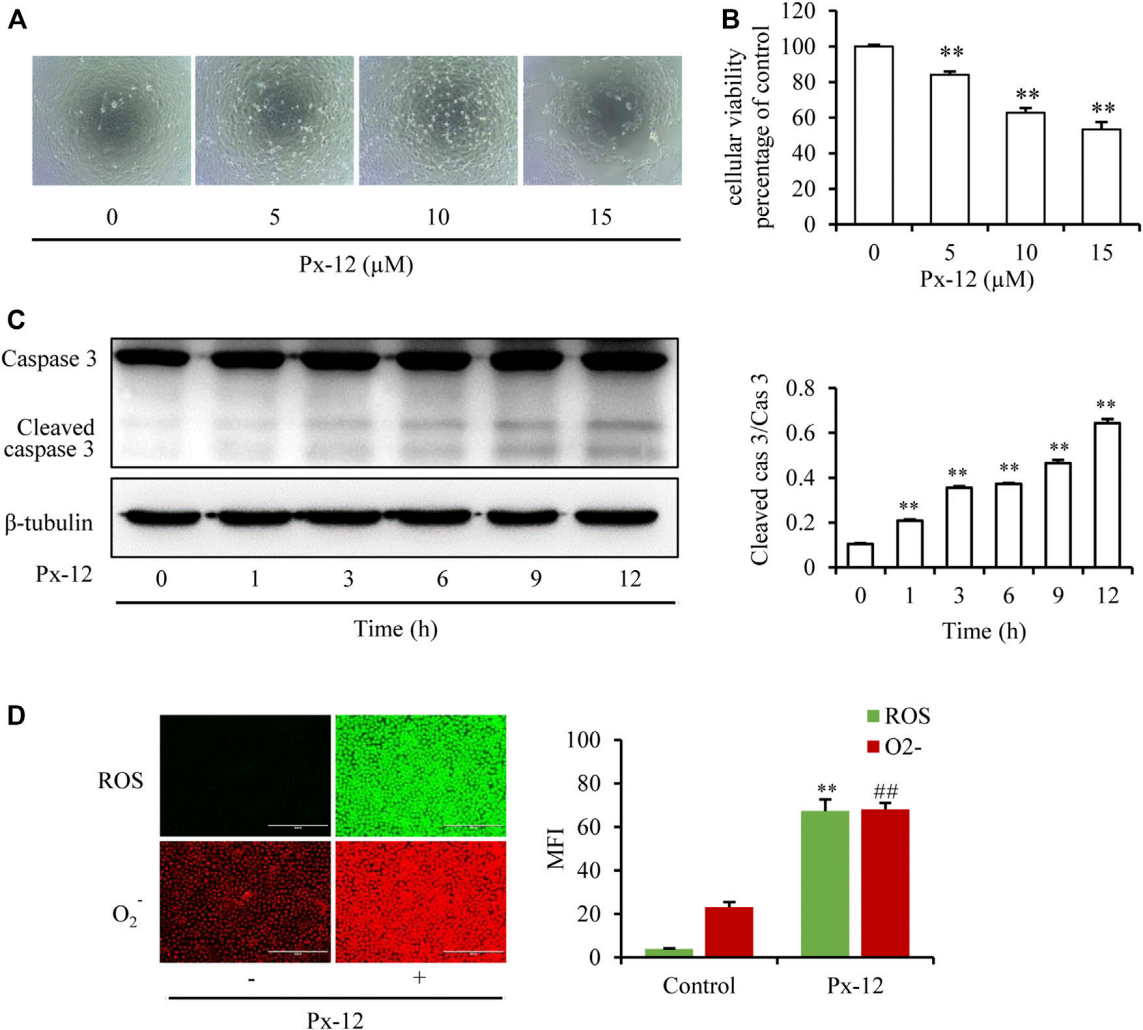
Inhibition of thioredoxin 1 induces renal tubular cell oxidative injury. **(A)** Effects of Px-12 on cellular morphology. NRK-52E cells in 96-well plates were exposed to different concentrations of Px-12 (0, 5, 10, and 15 µM) for 24 h. Cell morphology was determined using an inverted microscope (×100). **(B)** Change of cellular viability induced by Px-12. NRK-52E cells in 96-well plates were insulted with different concentrations of Px-12 (0, 5, 10, and 15 µM) for 24 h. Then, cell viability was tested using a Cell Counting Kit-8 (CCK-8) assay. Data are expressed as the percentage of living cells vs. the untreated control (mean ± SD, *n* = 4). *p < 0.01 vs. the control. **(C)** Px-12 triggers apoptosis. NRK-52E cells in 96-well plates were exposed to different concentrations of Px-12 for 24 h. Then, cells were lyzed, and total protein was extracted. Caspase-3 and cleaved caspase-3 are detected via western blot analysis. Densitometric analysis of cleaved caspase-3 is shown on the right (mean ± SD, *n* = 3; **p < 0.01 vs. the control). **(D)** Implications of Px-12 on reactive oxygen species (ROS)/O_2_^−^ generation. NRK-52E cells in six-well plates were exposed to Px12 (10 µM) for 1 h, then incubated with a ROS/O_2_^−^ probe for 30 min. Images have been acquired using an inverted fluorescence microscope. Mean fluorescence intensity is shown on the right (mean ± SD, *n* = 3; **p < 0.01 vs. the control, ##*p* < 0.01 vs. Px-12 in the control.).

It was first confirmed whether inhibition of thioredoxin 1 led to oxidative injury in renal tubular epithelial cells. To mimic the *in vitro* conditions of thioredoxin 1 deficiency in tubules, an inhibitor of thioredoxin, Px-12, was employed to induce NRK-52E cell oxidative injury. As shown in Figures 1A,B, Px-12 triggers NRK-52E cell injury, as evidenced by detachment from the bottom of the culture dish, changes in morphology, and loss of viability in a concentration-dependent manner. Caspase-3 is a characteristic cell apoptosis protein that is greatly increased in the middle-advanced stage of apoptosis (Bernard et al., 2019). Addition of Px-12 significantly increased the expression of cleaved caspase-3 after incubation for 1 h (Figure 1C). Thioredoxin 1 is an important component of the redox signal transduction system, which scavenges intracellular ROS to alleviate apoptosis. Indeed, inhibition of thioredoxin 1 enhanced production of ROS and O_2_- (Figure 1D). These results suggested that inhibition of thioredoxin 1 induced oxidative injury and subsequently triggered apoptosis in renal tubular cells.

### Inhibition of the Gap Junction Alleviated Px-12-Induced Cell Oxidative Injury

**FIGURE 2:**
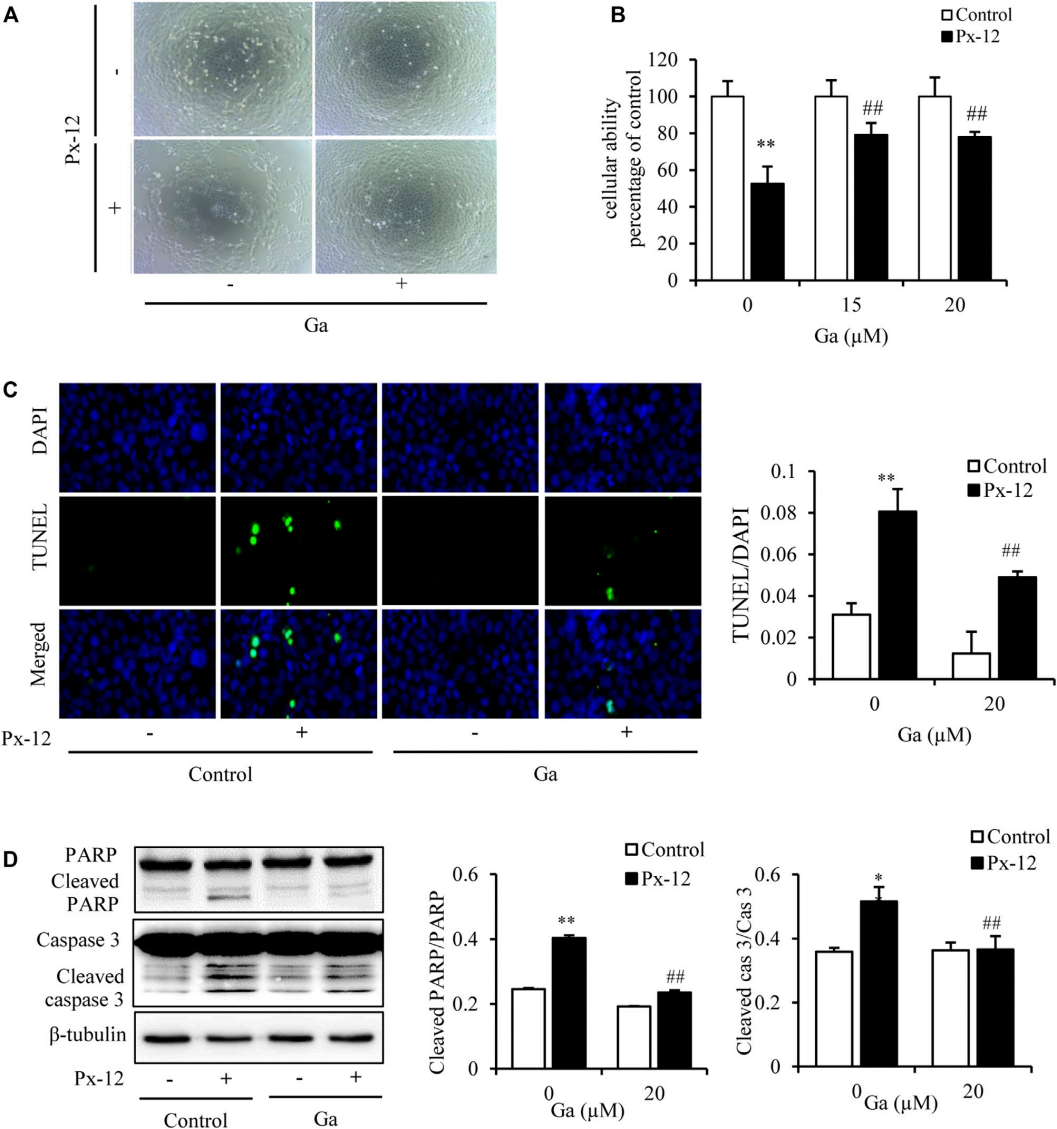
Inhibition of the gap junction alleviates Px-12-induced cell oxidative injury. **(A)** The role of 18α-glycyrrhetinic acid (Ga) on morphological changes triggered by Px-12. NRK-52E cells were pretreated with Ga (20 μM) for 1 h and then challenged with Px-12 (10 μM) for another 24 h. Cell morphology is shown, as determined using an inverted microscope (×100). **(B)** Effects of Ga on cell viability. NRK-52E cells were pretreated with Ga (15 and 20 μM) for 1 h and then challenged with Px-12 (10 μM) for another 24 h. Then, cell viability was evaluated using a Cell Counting Kit-8 (CCK-8) assay. Data are expressed as the percentage of living cells vs. the untreated control (mean ± SD, *n* = 6). *p < 0.01 vs. the control. ##*p* < 0.01 vs. Px-12 in the control. **(C)** Apoptosis staining of NRK-52E cells. NRK-52E cells in 96-well plate were pretreated with Ga (20 μM) for 1 h and then incubated with Px-12 for another 24 h. Then, apoptotic cells were evaluated using DT-mediated dUTP nick end labeling (TUNEL) and 4,6-diamidino-2-phenylindole (DAPI) staining (magnification, ×400). The ratio of positive cells is shown on the right (mean ± SD, *n* = 3; **p < 0.01 vs. the control, ##*p* < 0.01 vs. Px-12 in the control). **(D)** The role of Ga on NRK-52E cell apoptosis-related molecules. NRK-52E cells in six-well plates were pretreated with Ga (20 μM) for 1 h and then challenged with Px-12 for another 24 h. Cellular lysates were then analyzed by western blotting to detect poly (ADP-ribose) polymerase (PARP), cleaved PARP, caspase-3, and cleaved caspase-3. Densitometric analysis of cleaved PARP and caspase-3 are shown on the right (mean ± SD, *n* = 3; **p < 0.01 vs. the control, ##*p* < 0.01 vs. Px-12 in the control).

Our previous studies have shown that inhibition of the gap junction alleviates a variety of kidney cell injuries (Yan et al., 2012; Gao et al., 2015). As shown in the current study, the gap junction inhibitor Ga improved both Px-12-induced morphological changes of NRK-52E cells, and Px-12-elicited loss of cellular viability (Figures 2A,B). TUNEL/DAPI fluorescence staining is a routine method for detecting cell death(Li et al., 2019). As shown in Figure 2C, Ga alleviates Px-12-triggered NRK-52E cell death. Ga significantly inhibited Px-12-induced cleavage of caspase-3 and PARP, two markers of cell apoptosis, in NRK-52E cells, as shown by western blot analysis (Figure 2D). These data indicated that inhibition of the gap junction by Ga ameliorated Px-12-induced cell injury.

### JNK-Mediated Oxidative Stress Induced Cx43 in Renal Tubular Cells

**FIGURE 3:**
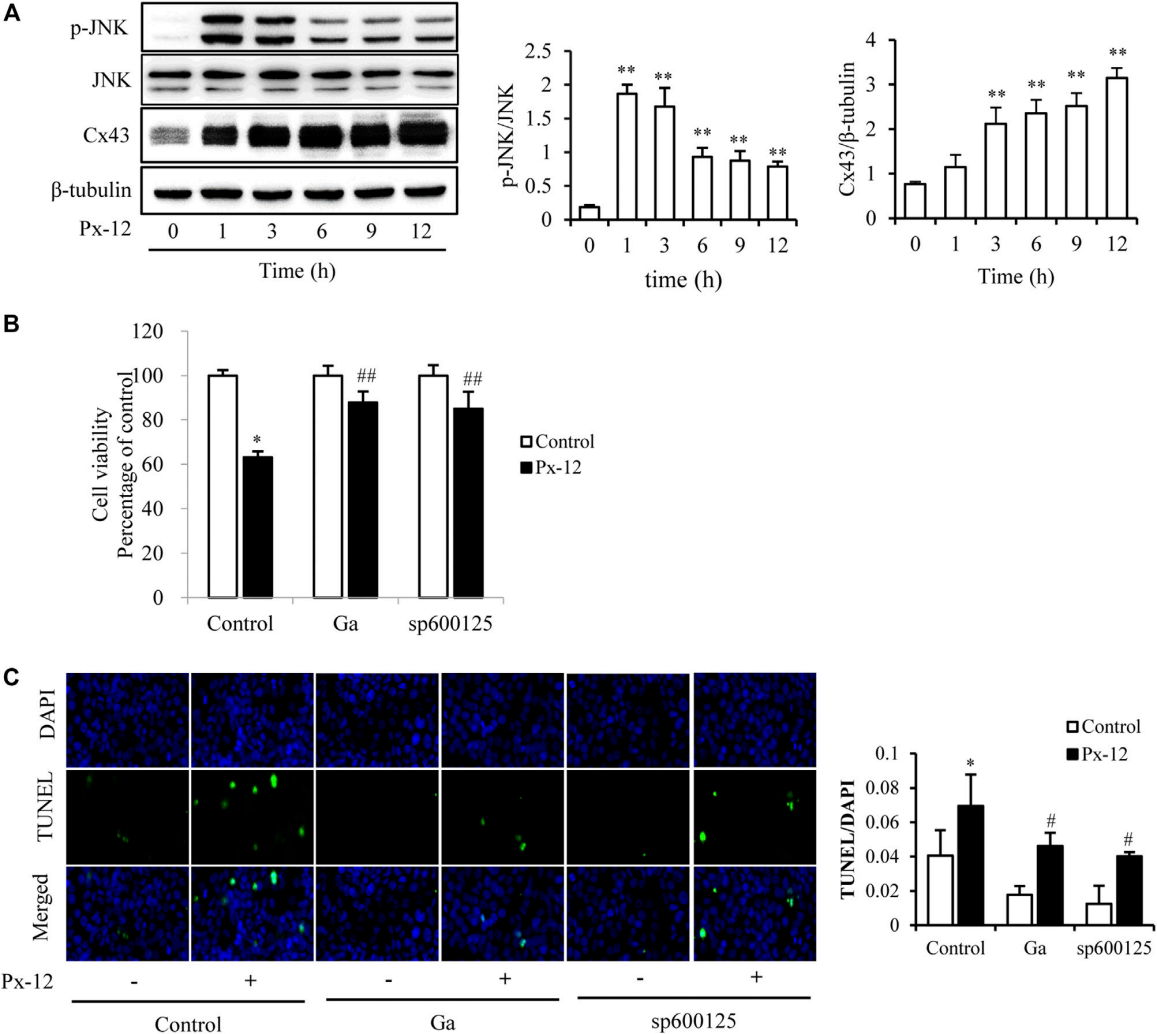
c-Jun N-terminal kinase (JNK)-mediated oxidative stress induces connexin 43 (Cx43). **(A)** Induction of JNK phosphorylation and expression of Cx43 by Px-12. NRK-52E cells in 6-well plates were incubated with Px-12 for 0, 1, 3, 6, 9, and 12 h separately. Cellular lysates were then analyzed by western blotting for phosphorylated JNK and Cx43 expression. Densitometric analysis of *p*-JNK and Cx43 are shown on the right (mean ± SD, *n* = 3; **p < 0.01 vs. the control, ##*p* < 0.01 vs. Px-12 in control). **(B)** Effects of 18α-glycyrrhetinic acid (Ga) and sp600125, an inhibitor of JNK, on Px12-induced cell injury. NRK-52E cells were pretreated with Ga (20 μM) and sp600125 (20 μM) for 1 h and then challenged with Px-12 for another 24 h. Then, cell viability was evaluated using a Cell Counting Kit-8 (CCK-8) assay. Data are expressed as the percentage of living cells vs. the untreated control (mean ± SD, *n* = 6). *p < 0.01 vs. the control. ##*p* < 0.01 vs. Px-12 in control. **(C)** Influence of sp600125 and Ga on NRK-52E cell apoptosis staining. NRK-52E cells in 96-well plate were pretreated with 18α-Ga and sp600125 for 1 h and then incubated with Px-12 for another 24 h. Then, apoptotic cells were stained via DT-mediated dUTP nick end labeling (TUNEL) and 4,6-diamidino-2-phenylindole (DAPI) (magnification, ×400). The ratio of positive cells is shown on the right (mean ± SD, *n* = 3; **p < 0.01 vs. the control, ##*p* < 0.01 vs. Px-12 in the control).

Since JNK mediates the induction of Px-12 and Cx43 in tumor cells (Li et al., 2016). Whether JNK was also involved in Px-12-induced renal tubular cell injury was tested. As shown in Figure 3A, Px-12 induces phosphorylation of JNK and subsequently the expression of Cx43. Meanwhile, both the inhibitor of the gap junction (Ga) and a JNK inhibitor (sp600125) improved Px-12-induced loss of cell viability (Figure 3B) and suppressed apoptosis (Figure 3C). We also tested the other two members of MAPK family, p38 and ERK. Ga didn’t influence the phosphorylation of p38 and ERK induced by Px-12 (Supplementary Material). The above result indicated that both JNK and Cx43 participated in Px-12-induced oxidative injury in renal tubular cells. Furthermore, JNK signaling was involved in the regulation of Px-12 on Cx43 in renal tubular cells.

### Reciprocal Modulation of Cx43 and JNK

**FIGURE 4:**
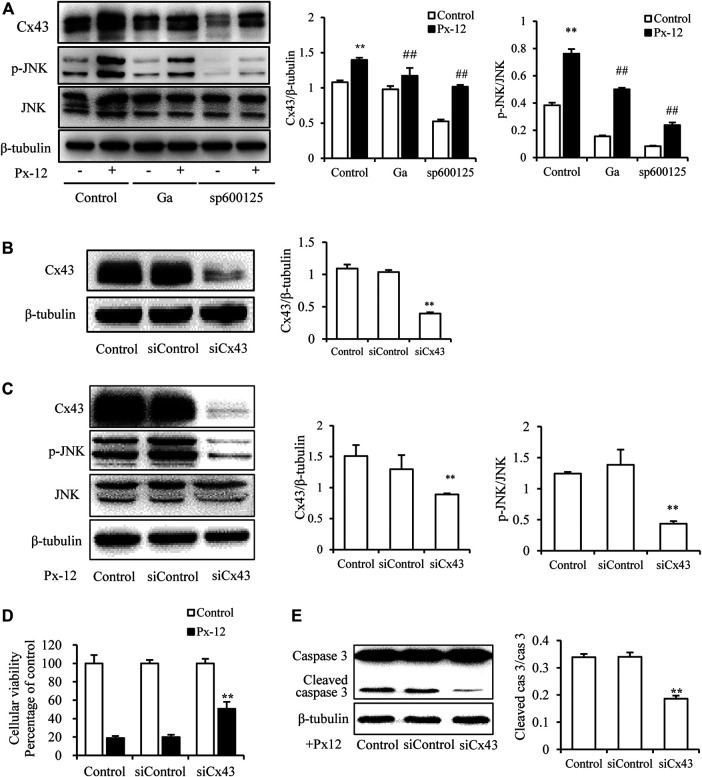
Reciprocal modulation of connexin 43 (Cx43) and c-Jun N-terminal kinase (JNK). **(A)** Effect of 18α-glycyrrhetinic acid (Ga) and sp600125 on JNK phosphorylation and expression of Cx43. NRK-52E cells in six-well plates were pretreated with Ga and sp600125 for 1 h and then challenged with Px-12 for another 1 h. Cellular lysates were then analyzed by western blotting for phosphorylated JNK and Cx43 expression. Densitometric analysis of *p*-JNK and Cx43 are shown on the right (mean ± SD, *n* = 3; **p < 0.01 vs. the control, ##*p* < 0.01 vs. Px-12 in the control). **(B)** NRK-52E cells were transfected with either Cx43 siRNA or siRNA control for 24 h. Cellular lysates were analyzed by western blotting for Cx43 expression. Densitometric analysis of Cx43 is shown on the right (mean ± SD, *n* = 3; **p < 0.01 vs. the siRNA control group). **(C)** Effect of Cx43 siRNA on JNK phosphorylation and Cx43 expression. The transfected or normal cells were incubated with Px-12 for 1 h separately. Then, cellular lysates were analyzed by western blotting for phosphorylated JNK and Cx43 expression. Densitometric analysis of *p*-JNK and Cx43 are shown on the right (mean ± SD, *n* = 3; **p < 0.01 vs. the control, ##*p* < 0.01 vs. Px-12 in the siRNA control group). **(D)** Effect of Cx43 siRNA on cell viability. The transfected or normal cells were incubated with Px-12 for 24 h. Then, cell viability was evaluated using a Cell Counting Kit-8 (CCK-8) assay. Data are expressed as the percentage of living cells vs. the untreated control (mean ± SD, *n* = 6). *p < 0.01 vs. Px-12 in normal cells. ##*p* < 0.01 vs. Px12 in the siRNA control group. **(E)** Effect of Cx43 siRNA on caspase-3 activation. The transfected or normal cells were incubated with Px-12 for 24 h. Then, cellular lysates were analyzed by western blotting for expression of cleaved caspase-3. Densitometric analysis of cleaved caspase-3 and caspase-3 are shown on the right (mean ± SD, *n* = 3; **p < 0.01 vs. Px-12 in the siRNA control group).

To further explore the regulation of JNK and Cx43 in Px-12-induced oxidative injury, the expression of Cx43 was downregulated using a JNK inhibitor (sp600125) and Cx43 siRNA transfection. As indicated in Figure 4A, sp600125 inhibits Px-12-induced phosphorylation of JNK and expression of Cx43. In contrast, Ga inhibited Cx43 expression and JNK phosphorylation (Figure 4A). Moreover, Cx43 downregulation by siRNA transfection inhibited the phosphorylation of JNK (Figures 4B,C). The improvement in Px-12-induced cell viability reduction (Figure 4D) and inhibition of caspase-3 (Figure 4E) by suppression of Cx43 further confirmed the role of Cx43 in renal tubular cell injury. These outcomes suggested a reciprocal modulation between Cx43 and JNK signaling, both underlying Px-12-induced oxidative injury.

### Cx43 Modulated Oxidative Stress by Regulating Thioredoxin 1

**FIGURE 5:**
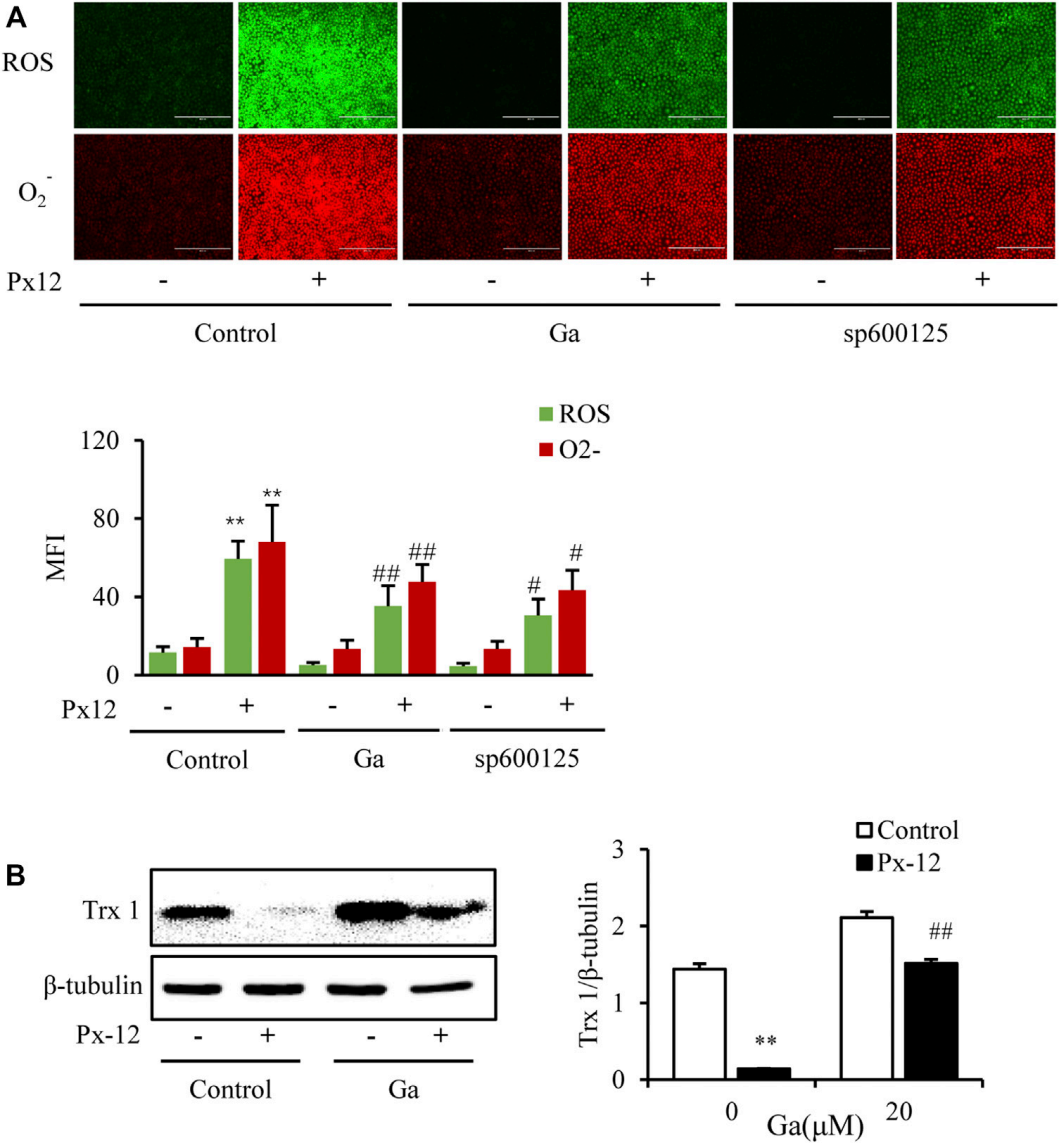
Connexin 43 (Cx43) inhibition suppresses oxidative stress by regulating thioredoxin 1. **(A)** Effect of 18α-glycyrrhetinic acid (Ga) and sp600125 on reactive oxygens species (ROS)/O_2_^−^ production. NRK-52E cells in a 96-well plate were pretreated with Ga and sp600125 separately for 1 h, challenged with Px-12 for another 1 h, and then incubated with a ROS/O_2_^−^ probe for 30 min. Images were captured using an inverted fluorescence microscope (×100). Mean fluorescence intensity is shown at the bottom (mean ± SD, *n* = 3; **p < 0.01 vs. the control, ##*p* < 0.01 vs. Px-12 in the control). **(B)** Effects of Ga on thioredoxin 1. NRK-52E cells in six-well plates were pretreated with Ga for 1 h and then challenged with Px-12 for another 1 h. Then, cellular lysates were analyzed by western blotting for the expression of thioredoxin 1. Densitometric analysis of thioredoxin 1, as determined by ImageJ, is shown on the right (mean ± SD, *n* = 3; **p < 0.01 vs. the control, ##*p* < 0.01 vs. Px-12 in the siRNA control group).

As mentioned above, Ga ameliorated Px-12-induced oxidative injury and significantly inhibited Px-12-induced over production of ROS and O_2_^−^ (Figure 5A). Since Cx43 regulates Txnip, the thioredoxin inhibitor, this indicated the possibility of indirect regulation of thioredoxin by Cx43. To determine the direct modulation of thioredoxin 1 by Cx43, the role of Ga on the thioredoxin 1 protein was evaluated. Ga indeed alleviated the inhibition of thioredoxin 1 by Px-12 (Figure 5B). These data indicated that the modulation of oxidative stress by Cx43 was ascribed to regulation of thioredoxin 1.

**FIGURE 6:**
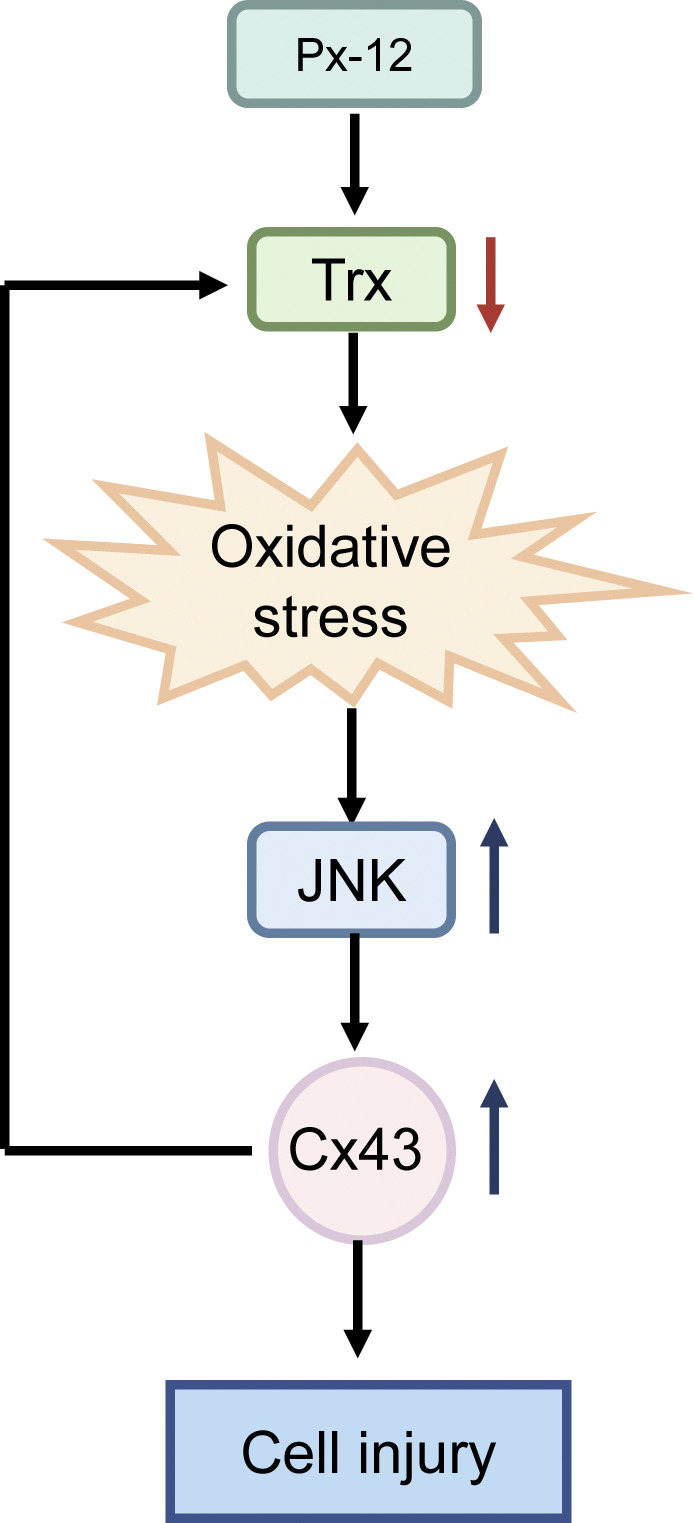
Schematic depiction of reactive oxygens species (ROS)-c-Jun N-terminal kinase (JNK)-connexin 43 (Cx43)-thioredoxin 1 signaling mechanisms involved in renal tubular cell redox signaling. Inhibition of thioredoxin 1 by Px-12 induces oxidative stress, JNK activation, and subsequent Cx43 expression. Suppression of Cx43 improves levels of thioredoxin 1 and inhibits ROS-JNK. ROS-JNK-Cx43-thioredoxin 1 are reciprocally regulated and participate in renal tubular oxidative injury.

## Discussion

In this study, it was first demonstrated that Cx43 was involved in thioredoxin 1 deficiency-induced oxidative renal tubular cell injury. Ga, a gap junction inhibitor, reduced oxidative injury by restoring levels of thioredoxin 1. The JNK inhibitor, sp600125, suppressed Px12-induced Cx43 expression. In contrast, Ga inhibited Px-12-induced JNK phosphorylation. Thus, JNK/MAPK, Cx43 and thioredoxin 1 signaling pathways were reciprocally regulated and participated in renal tubular oxidative injury (Figure 6).

Oxidative stress is one of the crucial mechanisms underlying the progression of CKD (Coppolino et al., 2018). Much progress has been made in the past decade with regard to defining oxidative stress. Although the field was traditionally defined as an imbalance of pro-oxidants and antioxidants, a large amount of attention has been paid to an imbalance between oxidants and antioxidants in favor of the oxidants, leading to a disruption of redox signaling and control, and/or molecular damage (Jones, 2006; Morita et al., 2019). This emphasized the critical role of redox signaling in specific conditions. The present study showed that inhibition of thioredoxin 1 subsequently elicited oxidative stress and apoptosis. Ga and the JNK inhibitor both significantly inhibited Px-12-induced cell apoptosis. Ga alleviated thioredoxin 1 that was suppressed by Px-12, and oxidative stress. Thus, it was revealed for the first time, that ROS-JNK-Cx43-thioredoxin 1 signaling specifically participated in renal tubular cell oxidative injury.

The thioredoxin system, which is composed of nicotinamide adenine dinucleotide phosphate (NADPH), thioredoxin reductase, and thioredoxin, is a key thiol antioxidant system defending against oxidative stress through its disulfide reductase activity regulating protein dithiol/disulfide balance (Koháryová and Kollárová, 2015). There are two thioredoxin systems in mammals, the cytosolic thioredoxin 1 system and the mitochondrial thioredoxin 2 system (Lu and Holmgren, 2014). Thioredoxin 1 has more cysteines than thioredoxin 2; thus, thioredoxin 1 plays a more important role in the redox process (Shinkai et al., 2012). Px-12 is an inhibitor of thioredoxin 1, causes redox signal transduction abnormalities, and eventually leads to oxidative injury (May et al., 2018). Txnip, an endogenous inhibitor of thioredoxin, binds to it and forms a redoxisome (Ji Cho et al., 2019), which exerts multiple functions in various biological processes. Inhibition of Cx43 promotes Txnip phosphorylation and degradation via extracellular signal-regulated kinase (ERK) (Gao et al., 2017) and indicates the important role of Cx43-ERK-Txnip signaling in renal oxidative injury. Moreover, it is possible that Cx43 regulates thioredoxin 1 indirectly by Txnip. Another question was whether Cx43 regulated thioredoxin 1 directly. The current study showed that the Cx43 inhibitor restored thioredoxin 1 inhibited by Px-12. This demonstrated that Cx43 regulated thioredoxin 1 both directly and indirectly.

ERK belongs to the MAPK family, which usually consists of ERK, JNK, and p38 kinase (Braicu et al., 2019). The MAPK signaling pathway is activated by multiple factors, such as growth factors, cytokines, pH, oxidative stress, and pressure stress (Gaestel, 2015). It is essential in regulating many cellular processes, including oxidative stress, inflammation, cell differentiation, and proliferation (Lake et al., 2016). To determine whether MAPKs were also involved in the effect of Cx43 on thioredoxin 1, ERK1/2, JNK, and p38 were tested. Interestingly, neither ERK nor p38 kinase was involved in role of Cx43 on thioredoxin 1 in renal cells. However, JNK participated in the regulation between Cx43 and thioredoxin 1 specifically in renal cells, leading to the question of how JNK regulated Cx43? JNK activation increases Cx43 expression at both the mRNA and protein levels (Li et al., 2013). In our previous study, upregulation of Cx43 increases sensitivity to Px-12-induced JNK activation and cell death in tumor cells (Li et al., 2016). Here, the role of the JNK-Cx43-thioredoxin 1 transduction pathway was further confirmed in renal redox signaling.

The mechanism by which Cx43 regulates oxidative stress is both channel dependent and channel independent (Yan et al., 2012). Under some conditions, Cx43 hemichannels are activated and open, which allows the exchange of small molecules into and out of the cytoplasm (Prakoura et al., 2018). The loss of some antioxidants results in the imbalance of anti- and pro-oxidants, such as glutathione and ATP (Fang et al., 2011; Chi et al., 2014). However, we have reported that there is no functional gap junction channel in podocytes and NADPH oxidase-mediated upregulation of Cx43 contributes to podocyte injury (Yan et al., 2012). In this study, a pivotal role for Cx43 in the regulation of thioredoxin 1 was demonstrated in a channel-independent manner.

In the current study, it was possible that the rescue of thioredoxin 1 by Ga might facilitate ROS delimitation and result in the suppression of dissociation of apoptosis signal-regulating kinase 1 (ASK1) from thioredoxin 1 and the downstream activation of JNK. ASK1 is known as mitogen-activated protein kinase 5 (MAP3K5) and is the upstream activator of p38 and JNK MAPK in response to a variety of stressors, such as oxidative stress, endoplasmic stress, and osmosis stress(Ichijo, 1997). This implied that there was probably a reciprocal interaction between Cx43 and the JNK pathway, and both markedly regulated redox signaling. Thus, thioredoxin 1 played a critical role in the modulation of ROS-JNK-Cx43-thioredoxin 1 redox signaling.

These findings have several significant implications. First, different oxidative conditions are ascribed to specific redox signaling dysregulation. Second, extracts from Chinese medicine herbs exert multiple functions under various circumstances. Third, regulation of both Txnip and thioredoxin 1 by Cx43 indicated the important role of Cx43 in modulation of oxidative stress mediated by the Txnip/thioredoxin redoxisome. Lastly, since renal tubular cells are a high metabolism cell with multiple functions, regulation of redox signaling is thought to be a therapeutic target.

Collectively, ROS-JNK-Cx43-thioredoxin 1 signaling was characterized in renal tubular oxidative stress. Regulation of Cx43 and thioredoxin 1 might be a novel target for the treatment of renal tubular disease.

## Data Availability Statement

The datasets presented in this study can be found in online repositories. The names of the repository/repositories and accession number(s) can be found in the article/Supplementary Material.

## Author Contributions

KG and DS designed the research. YZ, LG, PX, YZ, QW, WL, and QW performed the research. YZ, KG, and DS analyzed the data. QW participated in intellectual discussions. YZ and KG wrote the paper. All authors approved the final edited version.

## Funding

This study was financially supported by the National Natural Science Foundation of China (81673912 and 81873259 to KG) and the Priority Academic Program Development of Jiangsu Higher Education Institutions (Integration of Chinese and Western Medicine, to DS).

## Conflict of Interest

The authors declare that the research was conducted in the absence of any commercial or financial relationships that could be construed as a potential conflict of interest.
