# Supplementary figures and images for: Glycyrrhetinic Acid Protects Renal Tubular Cells against Oxidative Injury via Reciprocal Regulation of JNK-Connexin 43-Thioredoxin 1 Signaling
Source: Front Pharmacol. 2021 Feb 2;12:619567. doi: 10.3389/fphar.2021.619567 (PMC7884636; doi:10.3389/fphar.2021.619567)

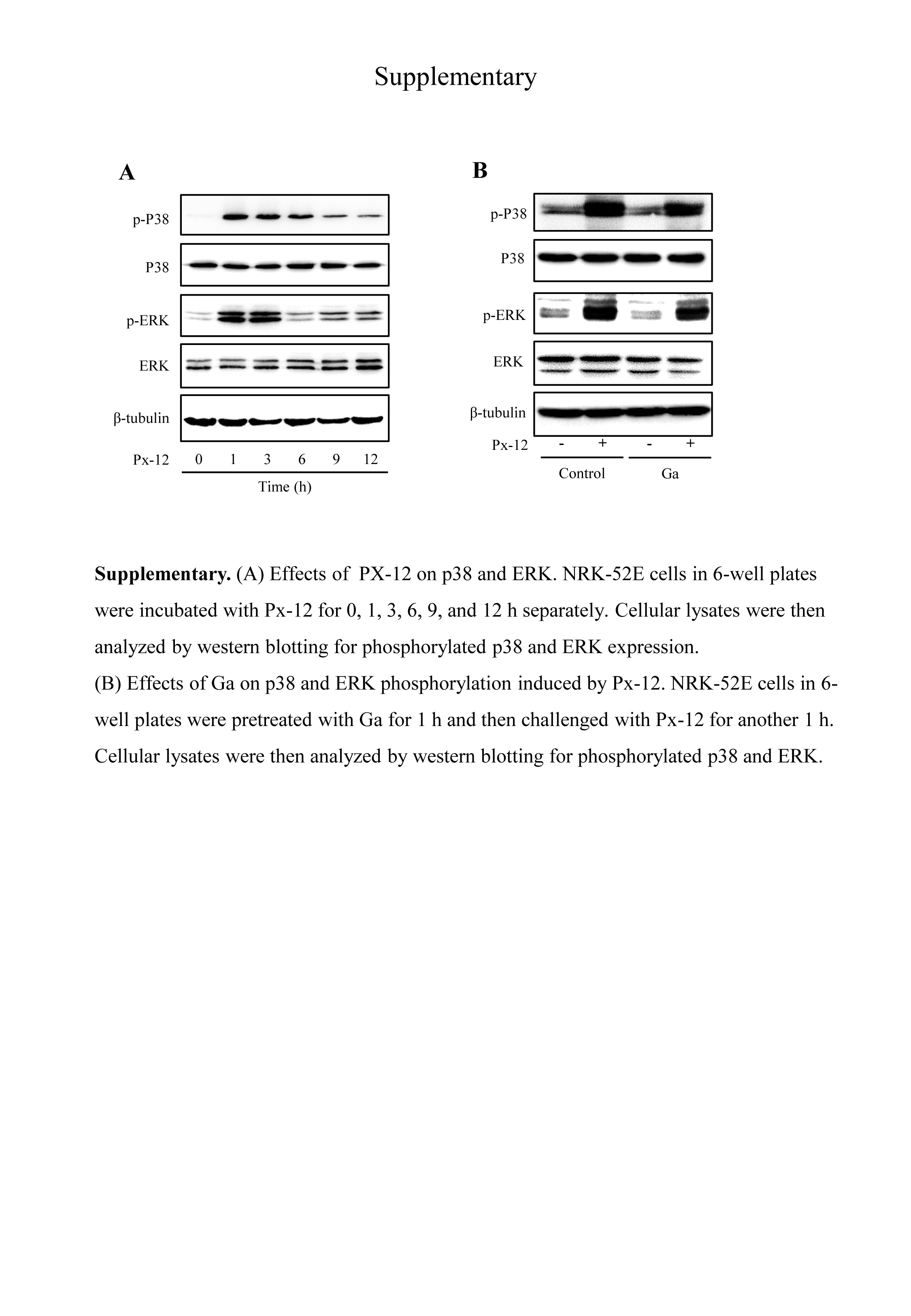

Supplement: Supplementary file 1 [file image1.jpeg]
